# Neoadjuvant camrelizumab combined with radiotherapy as a chemotherapy-sparing approach for resectable locally advanced esophageal squamous cell carcinoma: a phase II clinical trial (ESOCORT-NIRT)

**DOI:** 10.1186/s12916-026-04781-4

**Published:** 2026-03-09

**Authors:** Maohui Chen, Yizhou Huang, Shuliang Zhang, Cheng Chen, Taidui Zeng, Hongmu Li, Zhenyuan Yang, Chuanquan Lin, Wei Li, Benhua Xu, Chun Chen, Bin Zheng

**Affiliations:** 1 Department of Thoracic Surgery, Fujian Medical University Union Hospital, Fuzhou, China; 2 Key Laboratory of Cardio-Thoracic Surgery (Fujian Medical University), Fujian Province University, Fuzhou, China; 3 National Key Clinical Specialty of Thoracic Surgery, Fuzhou, China; 4 Clinical Research Center for Thoracic Tumors of Fujian Province, Fuzhou, China; 5 Department of Radiation Oncology, Fujian Medical University Union Hospital, Fuzhou, China; 6 Department of Thoracic Surgery Nursing, Fujian Medical University Union Hospital, Fuzhou, China

**Keywords:** Esophageal squamous cell carcinoma, Camrelizumab, Radiotherapy, Major pathologic response, Safety

## Abstract

**Background:**

Camrelizumab combined with radiotherapy may offer an effective neoadjuvant option for locally advanced esophageal squamous cell carcinoma while reducing chemotherapy-related toxicity. We assessed the efficacy and safety of this chemotherapy-sparing regimen.

**Methods:**

In this single-arm phase II trial, adults with resectable thoracic esophageal squamous cell carcinoma received camrelizumab 200 mg intravenously every 3 weeks for two cycles, concurrently with radiotherapy to 41.4 Gy in 23 fractions, followed by esophagectomy 4–8 weeks later. The primary endpoint was major pathologic response (≤ 10% residual viable tumor) among patients undergoing resection. Secondary endpoints were R0 resection rate, pathologic complete response, treatment-related adverse events, postoperative complications, and survival outcomes. Trial registration: ClinicalTrials.gov NCT05176002.

**Results:**

Twenty-five patients were enrolled; three did not undergo surgery (one radiographic complete response with refusal of surgery, one disease progression, and one withdrawal). Twenty-two patients (88%) had esophagectomy, all with R0 resection. Major pathologic response occurred in 12 of 22 patients (54.5%), including pathologic complete response in 8 (36.4%). No grade 3 or higher treatment-related adverse events were observed; the most frequent toxicities were low-grade radiation esophagitis, leukopenia, dermatitis, and hypothyroidism. Postoperative complications were low grade. At a median follow-up of 36 months, five recurrences were recorded, with survival comparable to historical standard neoadjuvant cohorts.

**Conclusions:**

Neoadjuvant camrelizumab plus radiotherapy showed low toxicity, pathologic tumor regression, and favorable postoperative outcomes in resectable esophageal squamous cell carcinoma. This chemotherapy-sparing approach may be an alternative for patients who are unsuitable for or intolerant of standard chemotherapy-based neoadjuvant regimens.

**Supplementary Information:**

The online version contains supplementary material available at 10.1186/s12916-026-04781-4.

## Summary box

### What is already known on this topic

Neoadjuvant chemoradiotherapy and chemoimmunotherapy improve outcomes in locally advanced esophageal squamous cell carcinoma but are often limited by chemotherapy-related toxicity and poor tolerance in some patients.

### What this study adds

This phase II study shows that neoadjuvant camrelizumab plus radiotherapy can achieve high rates of major pathologic response and R0 resection with very low rates of high-grade toxicity and generally mild postoperative complications.

### How this study might affect research, practice, or policy

These findings support further evaluation of chemotherapy-sparing neoadjuvant immunoradiotherapy regimens and suggest a potential treatment option for patients who are ineligible for standard chemotherapy-based strategies.

## Background

Esophageal cancer remains a major global health concern, with esophageal squamous cell carcinoma (ESCC) being the most common type encountered in East Asia [1–4]. The standard treatment for locally advanced ESCC involves neoadjuvant chemoradiotherapy (nCRT) followed by surgery, which significantly improves survival outcomes compared to surgery alone [5–7]. In recent years, evolving therapeutic approaches such as triplet chemotherapy represented by JCOG1109 and chemoimmunotherapy exemplified by ESCORT-NEO have demonstrated promising results and are increasingly shaping treatment paradigms [8, 9]. Furthermore, distant recurrence remains common, even after nCRT, and the addition of cytotoxic chemotherapy results in substantial toxicity. For example, large trials such as CROSS and NEOCRTEC5010 have reported high rates of distant metastasis (23–39%), despite improved survival, and most patients experience grade 3–4 hematologic and gastrointestinal adverse events [6, 7, 10]. This highlights the necessity for alternative strategies that improve efficacy, while minimizing treatment-related morbidity.

The advent of immune checkpoint inhibitors (ICIs) targeting programmed cell death protein 1(PD-1)/programmed death-ligand 1 (PD-L1) have transformed the treatment of ESCC [9, 11, 12]. Camrelizumab is a humanized anti–PD-1 monoclonal antibody that restores antitumor T-cell activity by blocking PD-1/PD-L1–mediated immune suppression and reshaping the tumor microenvironment toward immune activation. Several trials have investigated the combination of ICIs with chemotherapy or chemoradiotherapy to help improve pathological complete response (pCR) rates; however, this combination is associated with high toxicity [13–15]. For example, the phase I PALACE-1 trial (pembrolizumab + CROSS regimen) reported a 55.6% pCR, but with a 65% incidence of ≥ 3 grade adverse events [16]. Likewise, the phase III randomized trial ESCORT-NEO/NCCES01 reported higher pCR rates with camrelizumab plus chemotherapy, but also notable high-grade toxicities [9]. Together, these findings highlight both the potential benefit and the tolerability concerns regarding intensifying neoadjuvant therapy with ICIs.

Several studies have previously suggested that conventional radiation therapy functions by promoting systemic antitumor immunity, thereby reducing the risk of recurrence and improving patient survival [17]. Radiotherapy may synergize with PD-1 blockade by enhancing tumor antigen release and presentation, increasing T-cell infiltration, and potentially eliciting systemic immune effects. For other types of malignancies, immunotherapy combined with radiotherapy has demonstrated significantly better efficacy with an acceptable incidence of AEs [18, 19]. Indeed, relevant preclinical studies have shown that the effects of radiotherapy and immunotherapy are synergistic, rather than simply cumulative [20, 21]. Previous studies have shown that immunotherapy increases radiosensitivity in patients with ESCC, resulting in more effective control of local lesions and distant micrometastases, while radiotherapy enhances the effects of immunotherapeutic agents by upregulating PD-L1 expression in the tumor microenvironment [22, 23]. While this approach has been explored in other cancers, evidence in esophageal cancer remains limited. Therefore, we conducted the prospective, phase II ESOCORT-NIRT trial to evaluate the safety and efficacy of neoadjuvant camrelizumab plus concurrent radiotherapy alone. This study aims to determine if a chemotherapy-sparing approach can maintain high rates of pathological response while minimizing systemic toxicity, potentially offering a vital alternative for patients elderly or frail, and are therefore particularly vulnerable to chemotherapy-related toxicity.

## Methods

### Ethics statement

This single-arm phase II trial was conducted at Fujian Medical University Union Hospital (Fuzhou, China) following approval from the institutional ethics board (No. 2021YF040-01). All participants provided written informed consent to participate prior to enrollment. The trial was registered at ClinicalTrials.gov (NCT05176002) prior to patient recruitment, and was conducted in accordance with the tenets of the Declaration of Helsinki and Good Clinical Practice guidelines (Additional file 1. Study Protocol; Additional file 2: CONSORT list).

### Study design and oversight

The study was designed following Simon’s optimal two-stage design in order to assess the efficacy of the neoadjuvant regimen while allowing for early stopping if the treatment effect was deemed insufficient. In the first stage, 12 patients were enrolled. If ≤ 2 patients achieved major pathological response (MPR) in the first stage, the regimen would be considered not promising, and the trial would be halted owing to futility. Because more than 2 patients achieved MPR in stage I, the study proceeded to the second stage, enrolling an additional 13 patients (for a total planned sample size of 25). Meeting the primary efficacy endpoint required that at least 8 of 25 patients achieved MPR by the end of stage II; if this condition is met, the regimen would be deemed worthy of further investigation.

This two-stage design provided 90% power to declare the treatment effective if the true MPR rate was adequately high, while controlling the type I error at 10% (one-sided) and type II error at 20%. Throughout the study, patient safety, adverse events, and treatment feasibility were closely monitored by the investigators and reviewed in real time by a multidisciplinary team (MDT). This interim analysis allowed the trial to be stopped early if excessive toxicity emerged, or if the probability of meeting the primary efficacy endpoint was deemed too low.

### Patients

The eligibility criteria were as follows: adults aged 18–75 years old with histologically confirmed ESCC of the thoracic esophagus, classified as clinical stage T2–3 N0 M0 or T2–3 N + M0 (AJCC 8th edition), with an Eastern Cooperative Oncology Group (ECOG) performance status of 0 or 1. Key exclusion criteria included any prior anticancer therapy, active autoimmune disease, chronic systemic corticosteroid use (> 10 mg prednisone daily within 14 days), significant uncontrolled comorbidities (e.g., unstable cardiac disease), a history of interstitial lung disease or prior pneumonitis, active hepatitis B/C infection with high viral load, or any condition that investigators judged could compromise patient safety or protocol compliance. All patients were required to have adequate organ function for enrollment. The complete inclusion and exclusion criteria are available in our previously published trial protocol [24].

### Pretreatment workup and staging

All patients underwent comprehensive pretreatment evaluations and staging. Baseline assessments included physical examination, standard laboratory tests, and imaging studies. Contrast-enhanced computed tomography (CT) of the neck (if clinically indicated), chest, and abdomen was conducted for staging, along with esophagogastroduodenoscopy and endoscopic ultrasound for tumor assessment. Cervical ultrasonography was performed to evaluate cervical lymph nodes. Pulmonary function tests and echocardiography were conducted to assess each patient’s cardiopulmonary fitness for therapy. Positron emission tomography (PET)/CT was recommended for metabolic staging when available, although this was not mandatory. If airway involvement was suspected based on tumor location, bronchoscopy or endobronchial ultrasound was conducted to exclude tumor invasion of the trachea or bronchi. These investigations ensured accurate clinical staging prior to treatment initiation.

### Treatment

#### Neoadjuvant immunoradiotherapy (nIRT)

All patients received two cycles of camrelizumab (a PD-1 inhibitor) combined with radiotherapy as neoadjuvant therapy. Camrelizumab 200 mg was administered intravenously on day 1 of each of two 21-day cycles. Neoadjuvant radiotherapy was delivered 5 days per week using intensity-modulated radiotherapy (IMRT). The total prescribed dose was 40.0–41.4 Gy, administered in 23 fractions of 1.8 Gy. The gross tumor volume (GTV) encompassed the primary esophageal tumor and any involved regional lymph nodes; the clinical target volume (CTV) included the GTV plus a 3 cm margin in the cranial and caudal directions along the esophagus (to cover any potential microscopic spread), as well as the involved nodal regions; the planning target volume was defined as the CTV with an additional 5 mm margin to account for setup variability and respiratory motion.

#### Surgery

Approximately 4–8 weeks following the completion of neoadjuvant therapy, patients who showed no disease progression or new metastases, and who remained surgical candidates, were scheduled for esophagectomy. Minimally invasive esophagectomy was conducted whenever feasible; otherwise, an open transthoracic approach was applied. The surgical procedure typically followed either a McKeown (three-incision) or an Ivor Lewis (two-incision) esophagectomy, with two-field (thoracoabdominal) lymph node dissection routinely conducted, and three-field dissection (adding cervical nodes) conducted only in select cases. The specific operative technique (open vs. minimally invasive, two-field vs. three-field) was determined by the MDT based on the tumor location and patient factors. We defined the time-to-surgery as the interval from completion of neoadjuvant therapy to the date of esophagectomy (in weeks). Postoperative adjuvant therapy was not administered per protocol; any consideration of adjuvant treatment was individualized based on postoperative pathology, and was decided through multidisciplinary discussion.

### Assessments and endpoints

Baseline staging procedures and pathological confirmation were completed as described above. Tumor response following neoadjuvant therapy was evaluated by imaging approximately 4 weeks after NIRT, according to the Response Evaluation Criteria in Solid Tumors (RECIST) version 1.1 [25]. All patients who proceeded to surgery ultimately underwent complete tumor resection and pathological evaluation of the surgical specimen. The primary endpoint was the MPR rate, defined as the proportion of resected cases showing ≤ 10% residual viable tumor in the esophagectomy specimen (MPR is defined to be inclusive of patients achieving pCR). To ensure the robustness of this assessment, two gastrointestinal pathologists independently reviewed all resected specimens. If discrepancies occurred between the two gastrointestinal pathologists, a senior pathologist adjudicated the final assessment. These pathologists were blinded to the patients’ clinical information and pretreatment radiographic response. MPR was assessed among patients who completed the full neoadjuvant protocol and surgery. Secondary efficacy endpoints included the R0 resection rate (percentage of patients achieving microscopically margin-negative resection) and the pathological complete response (pCR) rate. The pCR was defined as no viable tumor cells detected in both the resected esophagus and sampled lymph nodes (ypT0N0). Safety endpoints included the incidence of adverse events (AEs) related to neoadjuvant therapy, graded according to the Common Terminology Criteria for Adverse Events (CTCAE) version 5.0, as well as the rates of postoperative complications and 30-day postoperative mortality, classified according to the Clavien–Dindo system [13, 26, 27].

Following completion of therapy and surgery, patients entered a structured follow-up program. Overall survival (OS) was defined as the time from the start of neoadjuvant therapy until death from any cause. Event-free survival (EFS) was defined as the time from the start of neoadjuvant therapy to the first occurrence of disease progression, tumor recurrence, or death from any cause. Posttreatment follow-up visits were conducted every 3 months for the first 2 years, then every 3–6 months for the next 3 years, and annually thereafter. At each follow-up, clinical evaluation and imaging (such as CT scans) were conducted to monitor for tumor recurrence or metastasis. In addition, tumor PD-L1 expression was assessed on resected specimens using the combined positive score (CPS), calculated as the number of PD-L1–positive cells (including tumor cells, lymphocytes, and macrophages) divided by the total number of viable tumor cells, multiplied by 100, and expressed as a percentage.

### Post hoccomparison with historical data

To contextualize outcomes from the nIRT cohort, we conducted a post hoc comparative analysis against historical treatments from our center. As the reference population, we used a previously published cohort of 182 patients who received either neoadjuvant immunochemotherapy (nICT) or nCRT [28]. The analysis first compared neoadjuvant treatment-related adverse event (TRAE) rates across the three groups (nIRT vs. nICT vs. nCRT).

To minimize any confounding from baseline imbalances, we applied inverse probability of treatment weighting (IPTW) based on propensity scores for the three treatment assignments (nIRT, nICT, nCRT). These scores were estimated using a multinomial logistic regression including tumor location (site), ECOG performance status, tumor location, and clinical disease stage as prespecified covariates. The inclusion of specific T and N stages was prioritized as they are critical independent determinants of both pathological response and long-term survival in ESCC. Stabilized weights were computed with the ipwpoint function in the R package ipw (version 1.2). Covariate balance was assessed in the weighted sample using standardized mean differences (SMDs), and the propensity model was iteratively refined (adding/removing polynomial terms and two-way interactions) until acceptable balance was achieved. After weighting, the baseline characteristics were well balanced, with SMDs < 0.25 for all included covariates.

In the IPTW-adjusted population, we generated covariate-adjusted survival curves and cumulative incidence estimates using Kaplan–Meier methods to compare outcomes between the study NIRT cohort and the historical nICT/nCRT cohorts. Weighted log-rank tests were applied to assess differences in time-to-event endpoints. Collectively, this post hoc analysis enabled an adjusted comparison of the efficacy and safety of neoadjuvant camrelizumab plus radiotherapy versus historical nICT and nCRT approaches.

### Statistical analysis

The data cutoff for analysis was September 1, 2025. All 25 enrolled patients were included in the safety analyses (intention-to-treat population for toxicity). Pathological efficacy outcomes (MPR and pCR) were primarily assessed in the per-protocol (PP) population, defined as patients who completed the planned neoadjuvant therapy and proceeded to esophagectomy. To mitigate potential attrition bias, we additionally performed a supportive intention-to-treat analysis including all 25 enrolled patients, wherein any patient who did not undergo surgery was strictly classified as a non-responder for pathological endpoints.

Continuous variables such as age and tumor length are presented as median values with interquartile ranges (IQRs), whereas categorical variables are summarized as frequencies and percentages. No missing data were observed for the primary endpoint and key secondary outcomes; therefore, no imputation was performed. Given the small sample size and non-normal data distributions, group comparisons for continuous variables were conducted using the Mann–Whitney *U* test. Categorical variables were compared using Fisher’s exact test (or chi-square test when appropriate); for 2 × 2 contingency tables, two-tailed *p*-values were calculated as twice the one-tailed exact probability, while two-tailed exact *p*-values were reported for larger tables. The Kaplan–Meier method was applied to estimate time-to-event endpoints including OS and EFS, with corresponding 95% confidence intervals. Survival curves were compared using log-rank tests. A *p*-value < 0.05 (two-sided) was considered statistically significant for all analyses. All statistical analyses were performed using R software (version 4.4.2; R Foundation for Statistical Computing, Vienna, Austria).

### Role of the funding source

The sponsors of this study had no role in the study design, patient recruitment, data collection, data analysis, data interpretation, or writing of the manuscript. The decision to submit the article for publication was made by the investigators independently. The corresponding authors (C.C. and B.Z.) had full access to all the data in the study and took final responsibility for the content and publication submission.

## Results

### Patient characteristics and treatment delivery

**Fig. 1:**
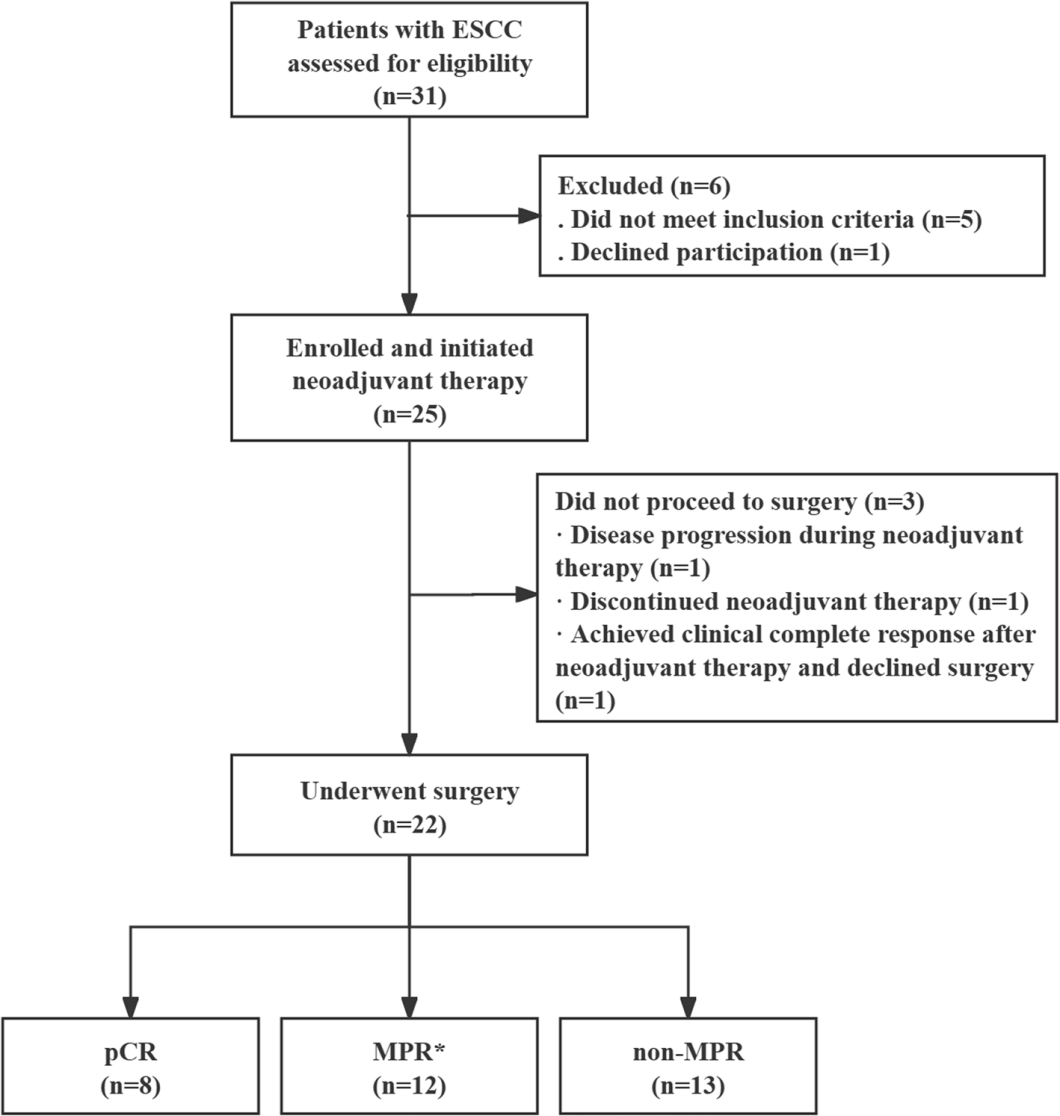
CONSORT diagram of the trial. * MPR is defined to be inclusive of patients achieving pCR

Between 2021 and 2023, 25 eligible patients with resectable locally advanced ESCC were enrolled and underwent nIRT (Fig. 1). The median age of this cohort was 63 years (range 49–75), and 80% of patients were male (Table 1). The baseline performance status was good: 20 patients (80%) had an ECOG score of 0, while 5 (20%) had ECOG 1. Most tumors were located in the middle thoracic esophagus (15 patients, 60%), with 28% in the distal third and 12% in the proximal third. The mean endoscopic tumor length was 4.5 ± 1.2 cm. Most patients (80%) showed clinical stage III disease at presentation, with 8% showing stage II and 12% stage IVA. Tumor differentiation was grade 1–2 in 72% of cases, and 28% of patients showed high PD-L1 expression (CPS ≥ 10).

**Table 1:** Baseline characteristics of the study population

	Overall (*n* = 25)
Age at enrollment (years)	
Mean ± SD	62.2 ± 6.9
Median (range)	63 (49–75)
Sex	
Male	20 (80%)
Female	5 (20%)
ECOG performance status	
0	20 (80%)
1	5 (20%)
Body mass index, kg/m^2^	
≥ 18.5	22 (88%)
< 18.5	3 (12%)
Tumor location (*n*, %)	
Proximal third	3 (12%)
Middle third	15 (60%)
Distal third	7 (28%)
Tumor length (mean ± SD, mm)	4.5 ± 1.2
Smoking status	
Former/current	8 (32%)
Never	17 (68%)
Tumor differentiation	
G1	10 (40%)
G2	8 (32%)
G3	4 (16%)
Gx	3 (12%)
Clinical T stage	
2	3 (12%)
3	22 (88%)
Clinical N stage	
0	1 (4%)
1	11 (44%)
2	10 (40%)
3	3 (12%)
AJCC clinical stage	
II	2 (8%)
III	20 (80%)
IVA	3 (12%)
PD-L1 statusc (*n*, %)	
CPS ≥ 10	7 (28%)
CPS < 10	14 (56%)
unknown	4 (16%)

Of the 25 enrolled patients, one developed radiographic disease progression during neoadjuvant therapy and discontinued the protocol to receive chemoradiotherapy, while one declined further neoadjuvant treatment and withdrew from the study. The remaining 23 patients completed the planned nIRT without any treatment interruptions or dose reductions. Radiotherapy was delivered as prescribed in all cases, and the full course of camrelizumab was completed in all patients. After nIRT, one patient achieved a complete response on imaging and endoscopy and declined esophagectomy. The remaining 22 patients proceeded to surgical resection as scheduled, with no unexpected surgical delays attributable to toxicity.

### Surgical and pathologic outcomes

**Fig. 2:**
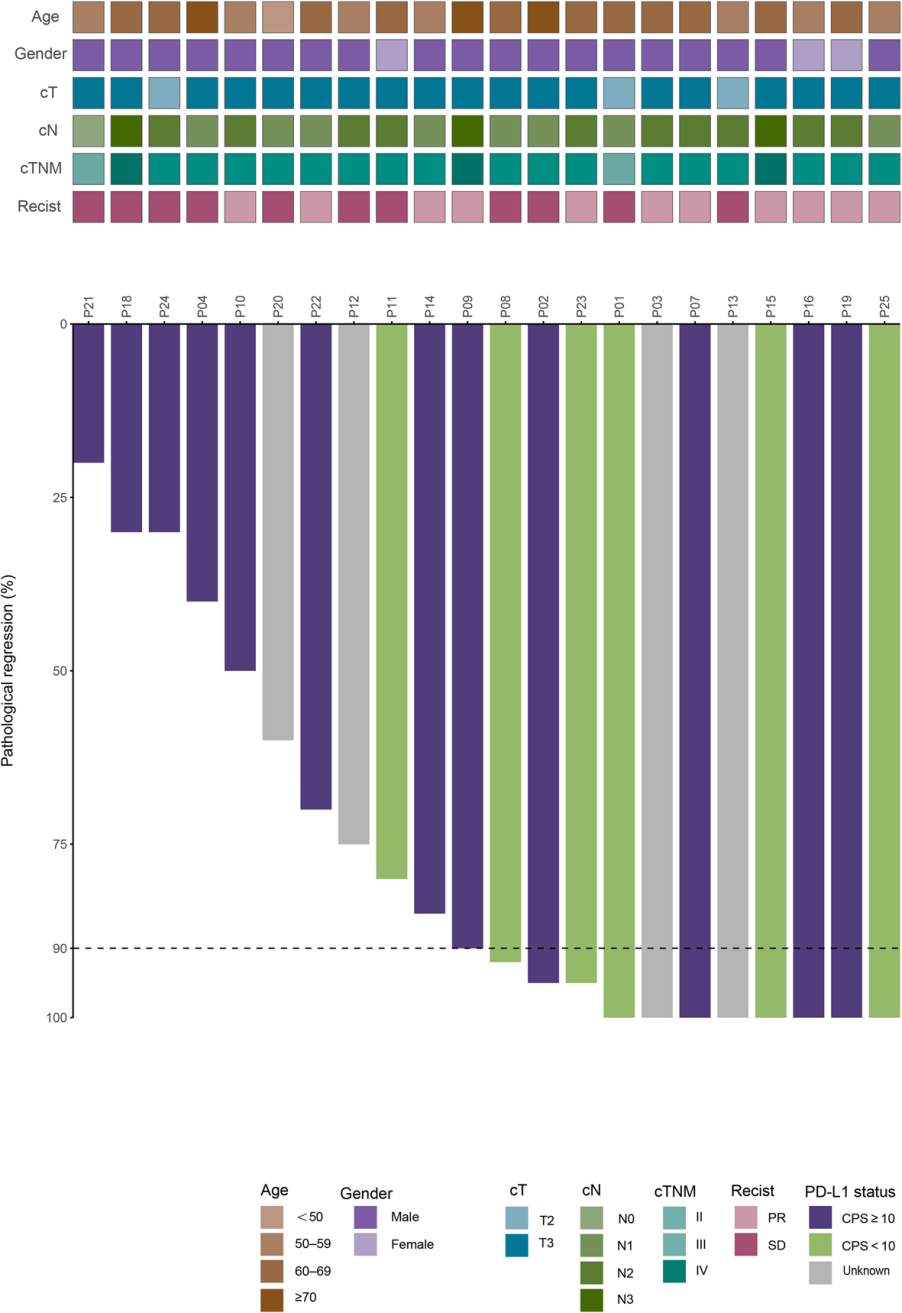
Pathological response after treatment with neoadjuvant Camrelizumab combined with radiotherapy. The gray horizontal line indicates the threshold for MPR patients. The clinical and pathological features are annotated for each patient, as is PD-L1 expression

Among the 22 patients who underwent esophagectomy with curative intent after NIRT, R0 resection was achieved in all cases (100%). In the PP population (*n* = 22), MPR occurred in 12 patients (54.5%), including 8 patients (36.4%) who achieved a pCR. In the supportive ITT analysis (*n* = 25), which accounts for the three patients who did not undergo surgery (one disease progression, one refusal, and one withdrawal), the MPR and pCR rates were 48.0% (12/25) and 32.0% (8/25), respectively (Fig. 2; Additional file 3: Table S1). Postoperative outcomes were favorable, with no perioperative deaths. Fourteen patients (63.6%) experienced postoperative complications, the vast majority of which were Clavien–Dindo grade 1–2 (Additional file 3: Table S2). One patient (4.5%) developed a grade 3 complication (an anastomotic leak requiring re-intervention); however, no grade 4 events occurred. The most common complications were transient recurrent laryngeal nerve palsy (6/22, 27.3%) and pneumonia (4/22, 18.2%), followed by arrhythmia (4/22, 18.2%) and anastomotic leakage (4/22, 18.2%). All complications were managed successfully.

### Safety and adverse events

All 25 patients were evaluable for TRAEs during neoadjuvant therapy (Table 2). No grade ≥ 3 TRAEs were observed, indicating an absence of severe acute toxicity (CTCAE v5.0 grade ≥ 3) from either camrelizumab or radiotherapy. The most common events were radiation esophagitis and hematologic suppression, all of which were grade 1–2: radiation esophagitis occurred in 9 patients (36%) and transient leukopenia in 7 (28%). Other commonly reported TRAEs (again, all confined to grade 1–2) were anorexia (24%, predominantly grade 1), elevated liver enzymes (16%), fatigue (16%), and pneumonitis (12%). One patient (4%) developed grade 1 hyperthyroidism attributable to immunotherapy. No treatment-related deaths occurred.

**Table 2:** Adverse events experienced by the study population

	Grade 1	Grade 2	Grade 3–4
Any adverse event	9 (36%)	8 (32%)	0 (0%)
Radiation esophagitis	7 (28%)	2 (8%)	0 (0%)
Leukopenia	3 (12%)	4 (16%)	0 (0%)
Anorexia	5 (20%)	1 (4%)	0 (0%)
Aminotransferase increased	3 (12%)	1 (4%)	0 (0%)
Fatigue	2 (8%)	2 (8%)	0 (0%)
Pneumonitis	2 (8%)	1 (4%)	0 (0%)
Nausea/vomiting	1 (4%)	1 (4%)	0 (0%)
Thrombocytopenia	2 (8%)	0 (0%)	0 (0%)
Diarrhea	2 (8%)	0 (0%)	0 (0%)
Creatinine increased	0 (0%)	1 (4%)	0 (0%)
Hyperthyroidism	1 (4%)	0 (0%)	0 (0%)

### Follow-up and recurrence

**Fig. 3:**
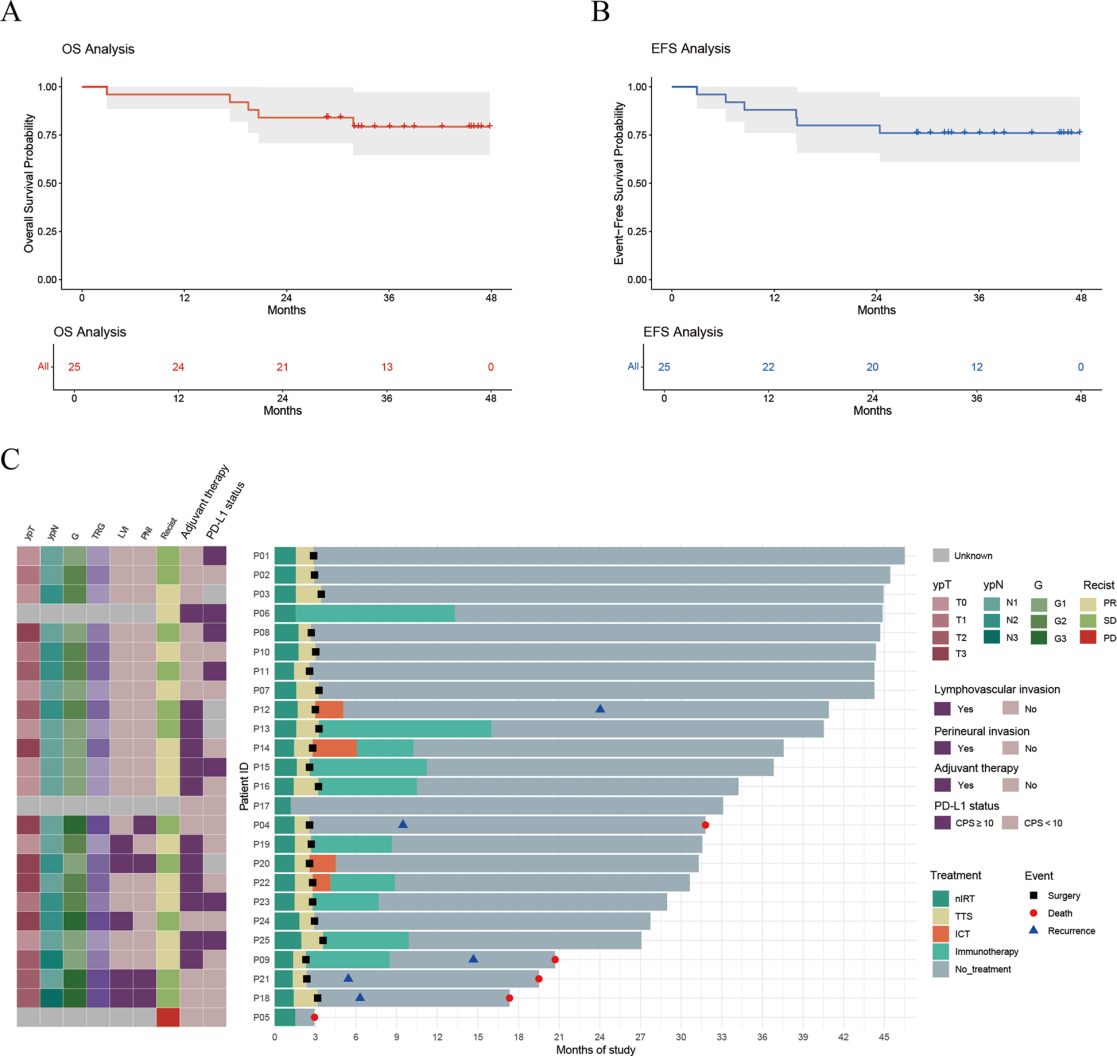
Neoadjuvant and adjuvant treatment regimens and patient-level follow-up (*n* = 25). **A**, **B** Kaplan–Meier curves for overall survival (OS; A) and event-free survival (EFS; B) among patients in the ESOCORT-NIRT trial who received treatment with neoadjuvant Camrelizumab plus radiotherapy followed by surgery. The median OS and EFS were not reached at the data cutoff. **C** Left, a dot plot annotating clinical and pathological features for each patient; right, a stacked bar chart showing the types and durations of treatments received. Symbols within each bar (circles or squares) denote key clinical events (e.g., death or surgery)

As of the data cutoff (September 1, 2025), the median follow-up time was 36 months (range, 3–48; Fig. 3). Five patients experienced recurrence, including two cases of locoregional nodal relapses, two cases of distant pulmonary metastases, and one case of local recurrence with multifocal liver and lung metastases. The median OS and EFS were not calculable at the time of analysis. In the intention-to-treat population (*n* = 25), the estimated 2-year EFS was 80% (20/25) and 2-year OS was 84% (21/25), with corresponding 3-year estimates of OS 79.3% and EFS 76%.

### Post hoccomparative analysis with historical cohorts

We conducted an exploratory post hoc comparison of the NIRT cohort with two historical cohorts treated at our institution: 88 patients who underwent standard nCRT and 94 patients who received nICT for locally advanced ESCC. Safety signals favored nIRT in sharp contrast with historical standards: the incidence rates of grade ≥ 3 TRAEs during the neoadjuvant phase were 0% with nIRT versus 14.8% with nCRT and 9.6% with nICT (Additional file 3: Fig. S1; Additional file 3: Table S3).

**Fig. 4:**
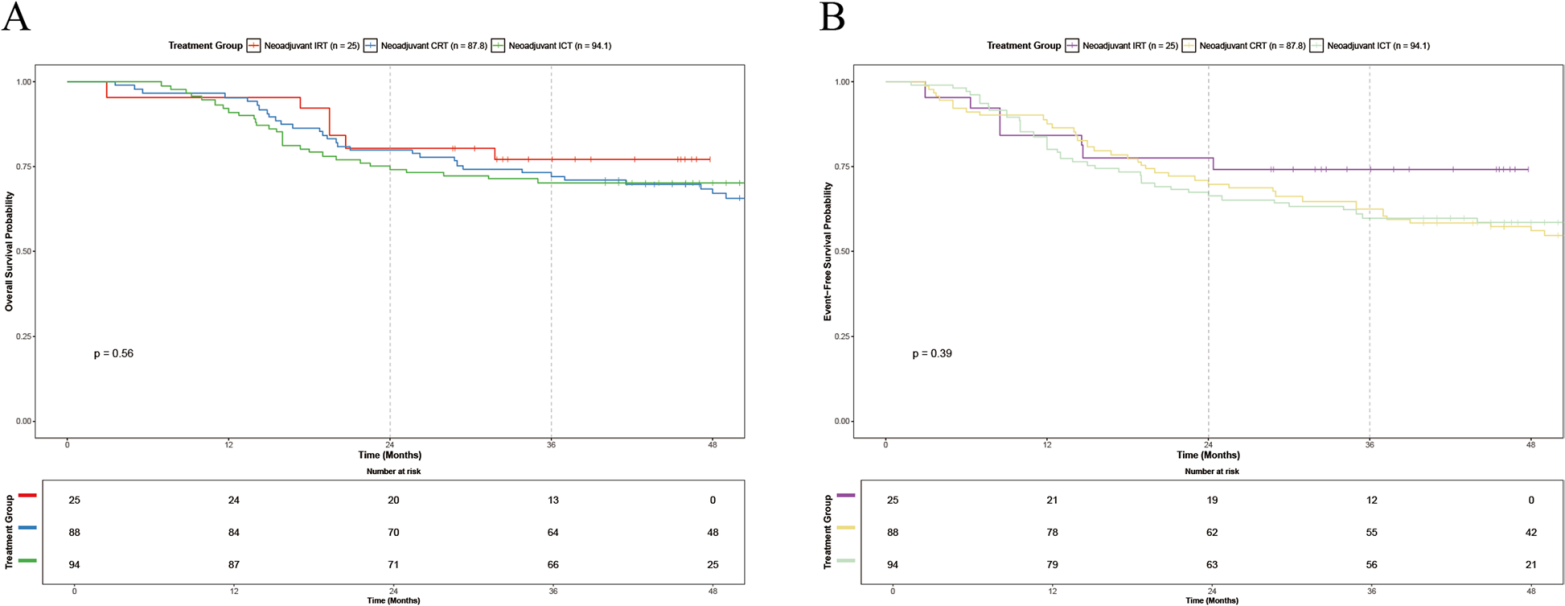
Kaplan–Meier curves of recurrence-free survival and overall survival in patients treated with neoadjuvant Camrelizumab combined with radiotherapy versus historical patients with chemoimmunotherapy or chemoradiotherapy after Inverse probability treatment weighting (IPTW) adjustment, respectively. The 2-year event-free survival and overall survival rate were estimated by Kaplan–Meier method

Although baseline characteristics differed among the three groups (e.g., the nCRT cohort was slightly younger than the nIRT cohort), IPTW was applied to minimize any confounding. Following IPTW adjustment, clinicopathologic variables were well balanced across groups (Additional file 3: Table S4). The Kaplan–Meier survival curves for the three treatments are shown in Fig. 4 and Additional file 3: Table S5. In the IPTW-adjusted analysis, the 2-year and 3-year EFS were 67.2% and 64.8% for NIRT, 70.0% and 62.5% for nCRT, and 66.0% and 59.3% for nICT. The 2-year and 3-year OS were 69.1% and 66.9% for NIRT, 79.9% and 72.0% for nCRT, and 74.0% and 70.0% for nICT. To provide effect-size estimates beyond weighted Kaplan–Meier curves, we additionally fitted IPTW-weighted Cox proportional hazards models with robust variance using NIRT as the reference. No statistically significant differences were observed in OS (nCRT vs NIRT: HR 0.956, 95% CI 0.37–2.471, *P* = 0.926; nICT vs NIRT: HR 0.872, 95% CI 0.33–2.302, *P* = 0.782) or EFS (nCRT vs NIRT: HR 1.103, 95% CI 0.411–2.958, P = 0.845; nICT vs NIRT: HR 1.084, 95% CI 0.402–2.925, *P* = 0.873).

## Discussion

This phase II ESCORT-NIRT trial provides important evidence to suggest that camrelizumab combined with concurrent radiotherapy could be an effective neoadjuvant treatment regimen for locally advanced, resectable ESCC. Overall, we observed a high rate of pathological tumor regression, with an MPR rate of 54.5% and pCR of 36.4%. All patients who proceeded to surgery achieved R0 resection. Importantly, no grade ≥ 3 toxicities occurred during the neoadjuvant period, indicating minimal severe hematologic or systemic adverse reactions. These findings indicate that omitting chemotherapy in the neoadjuvant setting did not compromise tumor downstaging; in fact, it markedly improved safety. In other words, NIRT maintained the high MPR/pCR rates comparable to those seen with chemoradiotherapy or chemoimmunotherapy regimens, while significantly improving tolerability. This is clinically meaningful for the many ESCC patients who are elderly or frail, and are therefore particularly vulnerable to chemotherapy-related toxicity.

Notably, the pCR rate of 36.4% in our study exceeds the ~ 28% reported with neoadjuvant camrelizumab plus chemotherapy in the ESCORT-NEO trial, and falls within the range achieved by neoadjuvant chemoradiotherapy in ESCC (for example, ~ 43% pCR in the NEOCRTEC5010 trial) [7, 9]. Moreover, all patients who underwent surgery in our study had complete (R0) resections, indicating that NIRT did not compromise resectability. Similarly, high pathological responses (pCR: 47.4%, MPR: 68.4%) were observed in another recent study of neoadjuvant immunotherapy investigating the PD-1 inhibitor toripalimab combined with radiotherapy in ESCC [29]. Collectively, these results indicate that in many cases, radiotherapy alone can synergize with PD-1 blockade to achieve effective tumor killing. This supports chemotherapy-free radio-immunotherapy as a potential alternative path in the neoadjuvant treatment paradigm for ESCC. However, it must be acknowledged that the high MPR/pCR observed here may reflect a substantial contribution from radiotherapy-driven local tumor control alone. While NIRT achieved MPR/pCR rates comparable to those of standard chemoradiotherapy or chemoimmunotherapy regimens, a high pathological response in the primary lesion does not necessarily equate to improved systemic disease control. These results indicate that radiotherapy can synergize with PD-1 blockade to achieve effective local tumor destruction, but the potential of this chemotherapy-sparing approach to eradicate subclinical micrometastases remains to be fully elucidated.

The safety profile of NIRT is another key advantage of this regimen. Previous neoadjuvant chemoradiotherapy trials, such as CROSS and NEOCRTEC5010, have established a survival benefit over surgery alone; however, pCR rates in modern ESCC studies are typically around 40%–50%, and most grade 3–4 toxicities in those regimens are attributable to chemotherapy [5, 7]. Recently, adding immunotherapy to neoadjuvant chemotherapy (termed “chemoimmunotherapy”) has shown promise: in the phase III ESCORT-NEO trial, the camrelizumab + chemotherapy arm achieved a pCR of 28% (versus 4.7% with chemotherapy alone), although grade ≥ 3 AEs occurred in ~ 30–34% of patients in both arms [9]. Similarly, adding PD-1 inhibitors to chemoradiotherapy yielded pCR rates of around 50–56% in single-arm studies (e.g., NEOCRTEC1901, PALACE-1), but at the cost of high toxicity (approximately 20% and 65% of patients experiencing grade ≥ 3 events in NEOCRTEC1901 and PALACE-1, respectively) [13, 16]. In contrast, our chemotherapy-free regimen achieved comparable MPR/pCR outcomes, but did not incur any grade ≥ 3 toxicities. This suggests that NIRT can capture the therapeutic benefit of immunotherapy augmentation, while markedly reducing severe toxicity.

Our preliminary survival and recurrence data are also encouraging. At a median follow-up of 36 months, five cases of recurrence were observed among the resected patients. Although our trial is single-arm and not powered for survival comparisons, these outcomes appear comparable to those of historical cohorts from our institution receiving standard nCRT or nICT. We did not detect any obvious differences in short-term overall or EFS between the NIRT cohort and those treated with these historical regimens, although longer follow-up is needed to confirm this finding. Nevertheless, this early observation of similar efficacy, combined with the reduced treatment-related morbidity, indicates that NIRT could offer a safer neoadjuvant approach without sacrificing oncologic outcomes. However, definitive conclusions about survival must await larger comparative studies.

In our cohort, all neoadjuvant TRAEs were classified grade 1–2 and manageable, with no grade 3–4 hematologic toxicities observed. This is in stark contrast to traditional chemotherapy-containing regimens, which commonly cause grade 3–4 neutropenia, leukopenia, and gastrointestinal toxicities [15, 30, 31]. The absence of any serious AEs indicates that NIRT may be particularly suitable for patients less tolerant of intensive chemotherapy, including those who are elderly, have marginal performance status, or carry comorbidities [32, 33]. Furthermore, the lack of any cases of severe toxicity meant that nearly all patients could proceed to surgery on schedule, without any significant delays. Postoperatively, most patients recovered well; complications were limited to Clavien–Dindo grade 1–2 events, and there were no surgery-related deaths. These findings indicate that NIRT did not compromise surgical feasibility or safety, and in fact may have facilitated timely surgery by avoiding chemo-related delays.

This study has several limitations. First, as a phase II, single-arm trial with a relatively small sample size, it lacked the statistical power to draw any definitive conclusions about survival outcomes; consequently, our results and the post hoc historical comparisons should be regarded as exploratory and hypothesis-generating, and must be interpreted cautiously due to potential residual confounding and selection bias. In addition, long-term endpoints (e.g., OS and EFS) remain immature, and whether the omission of chemotherapy might compromise systemic disease control in the long term requires further evaluation. Our protocol employed conventional radiotherapy fractionation rather than hypofractionation or dose escalation; whether alternative schedules could further potentiate immunotherapy effects therefore warrants investigation. Finally, future biomarker-driven studies (e.g., PD-L1 expression, tumor mutational burden, and circulating tumor DNA) are required to stratify patients who are most likely to benefit from a radio-immunotherapy strategy [34]. These limitations notwithstanding, the clear signals of safety and pathological efficacy observed here provide a strong rationale for further, adequately powered randomized phase III trials to validate these findings against chemotherapy-containing standards.

## Conclusions

In resectable ESCC, neoadjuvant camrelizumab combined with radiotherapy achieved a high rate of tumor regression (MPR 54.5%, pCR 36.4%), with an R0 resection of 100% and no ≥ 3 grade AEs. This chemotherapy-free radio-immunotherapy neoadjuvant strategy demonstrated feasibility and robust antitumor activity, offering a promising alternative for patients unsuitable for or intolerant of standard chemotherapy-based regimens. Its outstanding tolerability and efficacy profile support the necessity for further evaluation in randomized phase III trials to determine whether it could match or even surpass standard nCRT (and chemoimmunotherapy) in survival outcomes, particularly regarding long-term survival and systemic disease control, while substantially improving patient safety and quality of life.

## Supplementary Information

Additional file 1. Study Protocol.

Additional file 2. CONSORT checklist.

Additional file 3. Tables S1–S5 and Fig. S1. Table S1. Pretreatment clinical stage and posttreatment pathological stage. Table S2. Postoperative complications experienced by the study population. Table S3. Differences in treatment-related adverse events in patients who received neoadjuvant Camrelizumab combined with radiotherapy vs neoadjuvant chemoimmunotherapy or chemoradiotherapy. Table S4. Baseline patient characteristics in patients who received neoadjuvant Camrelizumab combined with radiotherapy vs neoadjuvant chemoimmunotherapy/chemoradiotherapy before and after IPTW adjustment. Table S5. Post hoc comparative analysis of 2-year and 3-year overall survival and event-free survival with historical data. Fig. S1. Comparison of treatment-related adverse events in neoadjuvant Camrelizumab combined with radiotherapyversus neoadjuvant chemoimmunotherapyand in nIRT versus neoadjuvant chemoradiotherapy. Grade 3 or more events were labeled in red color, Grade 1 or 2 events were labeled in blue.

## Data Availability

Individual data will be made available following publication by reasonable request to the corresponding author. The study protocol is available in the Additional file 1.

## Abbreviations

AEs: Adverse events
CPS: Combined positive score
CT: Computed tomography
CTCAE: Common Terminology Criteria for Adverse Events
CTV: Clinical target volume
ECOG: Eastern Cooperative Oncology Group
EFS: Event-free survival
ESCC: Esophageal squamous cell carcinoma
GTV: Gross tumor volume
ICIs: Immune checkpoint inhibitors
IMRT: Intensity-modulated radiotherapy
IPTW: Inverse probability of treatment weighting
MDT: Multidisciplinary team
MPR: Major pathological response
nCRT: Neoadjuvant chemoradiotherapy
nICT: Neoadjuvant immunochemotherapy
nIRT: Neoadjuvant immunoradiotherapy
OS: Overall survival
pCR: Pathological complete response
PD-1: Programmed cell death protein 1
PD-L1: Programmed death-ligand 1
PET: Positron emission tomography
PP: Per-protocol
RECIST: Response Evaluation Criteria in Solid Tumors
SMDs: Standardized mean differences
TRAE: Treatment-related adverse event
