# Supplementary material for: Neoadjuvant camrelizumab combined with radiotherapy as a chemotherapy-sparing approach for resectable locally advanced esophageal squamous cell carcinoma: a phase II clinical trial (ESOCORT-NIRT)
Source: BMC Med. 2026 Mar 9;24:244. doi: 10.1186/s12916-026-04781-4 (PMC13085588; doi:10.1186/s12916-026-04781-4)

**Additional file 3**

Table S1. Pretreatment clinical stage and posttreatment pathological stage

| **Patient ID** | **Pre-treatment clinical stage TNM (stage group)** | | **Pathologic stage at resection TNM (stage group)** | | | **Major Pathologic Response (yes/no)** | **PCR** | **Pathologic downstaging (yes/no)** | **Recist** |
| --- | --- | --- | --- | --- | --- | --- | --- | --- | --- |
| P01 | cT2N1M0 | II | | ypT0N0M0 | I | yes | yes | yes | SD |
| P02 | cT3N1M0 | III | | ypT1aN0M0 | I | yes | no | yes | SD |
| P03 | cT3N2M0 | III | | ypT0N0M0 | I | yes | yes | yes | PR |
| P04 | cT3N1M0 | III | | ypT3N0M0 | II | no | no | yes | SD |
| P05 | cT3N1M0 | III | | NA | NA | NA | NA | NA | PD |
| P06 | cT3N1M0 | III | | NA | NA | NA | NA | NA | PR |
| P07 | cT3N2M0 | III | | ypT0N0M0 | I | yes | yes | yes | PR |
| P08 | cT3N1M0 | III | | ypT3N0M0 | II | yes | no | yes | SD |
| P09 | cT3N3M0 | IVA | | ypT2N2M0 | IIIB | yes | no | yes | PR |
| P10 | cT3N2M0 | III | | ypT1bN1M0 | IIIA | no | no | no | PR |
| P11 | cT3N2M0 | III | | ypT2N1M0 | IIIA | no | no | no | SD |
| P12 | cT3N2M0 | III | | ypT2N1M0 | IIIA | no | no | no | SD |
| P13 | cT2N2M0 | III | | ypT0N0M0 | I | yes | yes | yes | SD |
| P14 | cT3N1M0 | III | | ypT3N0M0 | II | no | no | yes | PR |
| P15 | cT3N3M0 | IVA | | ypT0N0M0 | I | yes | yes | yes | PR |
| P16 | cT3N2M0 | III | | ypT0N0M0 | I | yes | yes | yes | PR |
| P17 | cT3N1M0 | III | | NA | NA | NA | NA | NA | NA |
| P18 | cT3N3M0 | IVA | | ypT2N3M0 | IIIB | no | no | yes | SD |
| P19 | cT3N2M0 | III | | ypT0N0M0 | I | yes | yes | yes | PR |
| P20 | cT3N1M0 | III | | ypT3N1M0 | IIIB | no | no | no | SD |
| P21 | cT3N0M0 | II | | ypT2N0M0 | I | no | no | yes | SD |
| P22 | cT3N1M0 | III | | ypT3N0M0 | II | no | no | yes | PR |
| P23 | cT3N2M0 | III | | ypT1bN1M0 | IIIA | yes | no | no | PR |
| P24 | cT2N2M0 | III | | ypT3N1M0 | IIIB | no | no | no | SD |
| P25 | cT3N1M0 | III | | ypT0N0M0 | I | yes | yes | yes | PR |

Table S2**.** Postoperative complications experienced by the study population.

| Postoperative complications, n (%) | Grade 1 | Grade 2 | Grade 3 | Grade 4 |
| --- | --- | --- | --- | --- |
| All Postoperative complications | 8 (36.4) | 5 (22.7) | 1 (4.5) | 0 (0) |
| Pulmonary complications | 1 (4.5) | 4 (18.2) | 0 (0) | 0 (0) |
| Pneumonia | 0 (0) | 4 (18.2) | 0 (0) | 0 (0) |
| Pleural effusion | 1 (4.5) | 1 (4.5) | 0 (0) | 0 (0) |
| Pneumothorax | 0 (0) | 1 (4.5) | 0 (0) | 0 (0) |
| Respiratory failure | 0 (0) | 0 (0) | 0 (0) | 0 (0) |
| Anastomotic leakage | 0 (0) | 3 (13.6) | 1 (4.5) | 0 (0) |
| Vocal cord paralysis | 6 (27.3) | 0 (0) | 0 (0) | 0 (0) |
| Arrhythmia | 2 (9.1) | 2 (9.1) | 0 (0) | 0 (0) |
| Abnormal liver function | 2 (9.1) | 1 (4.5) | 0 (0) | 0 (0) |
| 30-day mortality | 0 (0) | 0 (0) | 0 (0) | 0 (0) |

* Complications were graded according to the Clavien-Dindo classification for surgical complicatio

Table S3**.** Differences in treatment-related adverse events in patients who received neoadjuvant camrelizumab combined with radiotherapy vs neoadjuvant chemoimmunotherapy or chemoradiotherapy.

| Adverse events, n (%) | NCRT (n=88) | | | NICT (n=94) | | NIRT (n=25) |
| --- | --- | --- | --- | --- | --- | --- |
|  | Grade 1-2 | Grade≥3 | Grade 1-2 | | Grade≥3 | Grade 1-2 |
| Leukopenia | 56 (63.6) | 7 (8.0) | 46 (48.9) | | 5 (5.3) | 7 (28.0) |
| Nausea or vomiting | 56 (63.6) | 2 (2.3) | 63 (67.0) | | 2 (2.1) | 2 (8.0) |
| Anorexia | 49 (55.7) | 2 (2.3) | 57 (60.6) | | 1 (1.1) | 6 (24.0) |
| Radiation esophagitis | 44 (50.0) | 2 (2.3) | 0 (0) | | 0 (0) | 9 (36.0) |
| Anemia | 34 (38.6) | 3 (3.4) | 32 (34.0) | | 2 (2.1) | 0 (0) |
| Thrombocytopenia | 27 (30.7) | 1 (1.1) | 24 (25.5) | | 0 (0.0) | 2 (8.0) |
| Fatigue | 22 (25.0) | 1 (1.1) | 24 (25.5) | | 2 (2.1) | 4 (16.0) |
| Pneumonitis | 22 (25.0) | 0 (0) | 4 (4.3) | | 0 (0) | 3 (12.0) |
| Aminotransferase increased | 18 (20.5) | 0 (0) | 20 (21.3) | | 1 (1.1) | 4 (16.0) |
| Diarrhea | 5 (5.7) | 0 (0) | 6 (6.4) | | 0 (0) | 2 (8.0) |
| Creatinine increased | 3 (3.4) | 0 (0) | 7 (7.4) | | 0 (0) | 1 (4.0) |
| Hyperthyroidism | 0 (0) | 0 (0) | 8 (8.5) | | 0 (0) | 1 (4.0) |

Table S4**.** Baseline patient characteristics in patients who received neoadjuvant camrelizumab combined with radiotherapy vs neoadjuvant chemoimmunotherapy/chemoradiotherapy before and after IPTW adjustment.

| Characteristic, n(%) | Before IPTW | | | After IPTW | | |
| --- | --- | --- | --- | --- | --- | --- |
|  | IRT(n=25) | CRT(n=88) | ICT(n=94) | IRT(n=25.3) | CRT(n=87.7) | ICT(n=94.1) |
| Age at enrollment-year, mean ± SD | 62.2 ± 6.9 | 58.8 ± 7.0 | 61.6 ± 6.6 | 63.4 ± 6.3 | 58.6 ± 6.8 | 61.6 ± 6.5 |
| Gender |  |  |  |  |  |  |
| Male | 20 (80.0) | 75 (85.2) | 70 (74.5) | 22.6 (89.2) | 74.8 (85.2) | 69.2 (73.5) |
| Female | 5 (20.0) | 13 (14.8) | 24 (25.5) | 2.7 (10.8) | 12.9 (14.8) | 24.9 (26.5) |
| Tumor location |  |  |  |  |  |  |
| Proximal third | 3 (12.0) | 13 (14.8) | 13 (13.8) | 5.6 (22.1) | 12.6 (14.4) | 13.3 (14.2) |
| Middle third | 15 (60.0) | 59 (67.0) | 55 (58.5) | 13.8 (54.7) | 55 (62.6) | 58.5 (62.2) |
| Distal third | 7 (28.0) | 16 (18.2) | 26 (27.7) | 5.9 (23.2) | 20.2 (23.0) | 22.2 (23.6) |
| Clinical disease stage |  |  |  |  |  |  |
| II | 3 (12.0) | 17 (19.3) | 17 (18.1) | 4.2 (16.5) | 15.5 (17.7) | 16.3 (17.3) |
| III | 22 (88.0) | 38 (43.2) | 48 (51.1) | 12.7 (50.1) | 44.6 (50.8) | 48 (51.0) |
| IV | 0 (0) | 33 (37.5) | 29 (30.9) | 8.4 (33.4) | 27.6 (31.5) | 29.8 (31.6) |
| ECOG |  |  |  |  |  |  |
| 0 | 20 (80.0) | 74 (84.1) | 82 (87.2) | 21.7 (85.6) | 74.3 (84.7) | 79.9 (84.9) |
| 1 | 5 (20.0) | 14 (15.9) | 12 (12.8) | 3.6 (14.4) | 13.4 (15.3) | 14.2 (15.1) |

**Table S5.** Post hoc comparative analysis of 2-year and 3-year overall survival and event-free survival with historical data.

| Treatment, n(%)  Gender | Event free survival | | Overall survival | |
| --- | --- | --- | --- | --- |
|  | 24-months | 36-months | 24-months | 36-months |
| NIRT | 67.2% | 64.8% | 69.1% | 66.9% |
| NCRT | 70.0% | 62.5% | 79.9% | 72.0% |
| NICT | 66.0% | 59.3% | 74.0% | 70.0% |

**Figure S1.** Comparison of treatment-related adverse events in neoadjuvant camrelizumab combined with radiotherapy (nIRT) versus neoadjuvant chemoimmunotherapy (nICT) and in nIRT versus neoadjuvant chemoradiotherapy (nCRT). Grade 3 or more events were labeled in red color, Grade 1 or 2 events were labeled in blue.


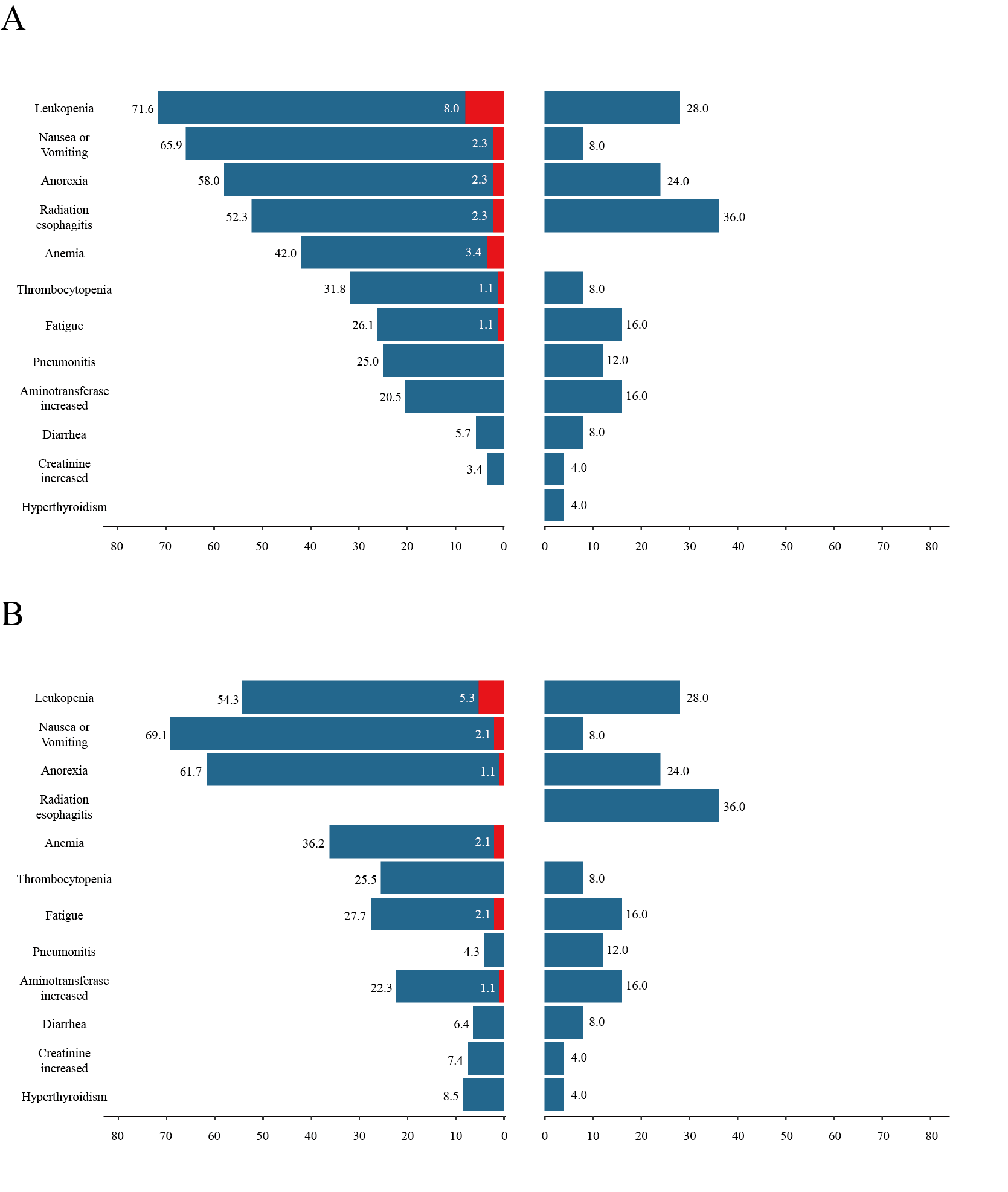

Supplement: Supplementary file 3 — Additional file 3. Tables S1–S5 and Fig. S1. Table S1. Pretreatment clinical stage and posttreatment pathological stage. Table S2. Postoperative complications experienced by the study population. Table S3. Differences in treatment-related adverse events in patients who received neoadjuvant Camrelizumab combined with radiotherapy vs neoadjuvant chemoimmunotherapy or chemoradiotherapy. Table S4. Baseline patient characteristics in patients who received neoadjuvant Camrelizumab combined with radiotherapy vs neoadjuvant chemoimmunotherapy/chemoradiotherapy before and after IPTW adjustment. Table S5. Post hoc comparative analysis of 2-year and 3-year overall survival and event-free survival with historical data. Fig. S1. Comparison of treatment-related adverse events in neoadjuvant Camrelizumab combined with radiotherapyversus neoadjuvant chemoimmunotherapyand in nIRT versus neoadjuvant chemoradiotherapy. Grade 3 or more events were labeled in red color, Grade 1 or 2 events were labeled in blue. [file 12916_2026_4781_MOESM3_ESM.docx]
